# Transforming growth factor beta1 targets estrogen receptor signaling in bronchial epithelial cells

**DOI:** 10.1186/s12931-018-0861-5

**Published:** 2018-08-30

**Authors:** L. Cody Smith, Santiago Moreno, Lauren Robertson, Sarah Robinson, Kristal Gant, Andrew J. Bryant, Tara Sabo-Attwood

**Affiliations:** 1 Department of Physiological Sciences, University of Florida, Gainesville, FL USA; 2 Center for Environmental and Human Toxicology, University of Florida, Gainesville, FL USA; 3 Department of Environmental and Global Health, Center for Environmental and Human Toxicology, University of Florida, Box 110885, 2187 Mowry Rd, Gainesville, FL 32611 USA; 4 Department of Medicine, University of Florida, Gainesville, FL USA

**Keywords:** Transforming growth factor beta1, Estrogen, Estrogen receptor, Lung, Fibrosis

## Abstract

**Background:**

Sex differences in idiopathic pulmonary fibrosis (IPF) suggest a protective role for estrogen (E2); however, mechanistic studies in animal models have produced mixed results. Reports using cell lines have investigated molecular interactions between transforming growth factor beta1 (TGF-β1) and estrogen receptor (ESR) pathways in breast, prostate, and skin cells, but no such interactions have been described in human lung cells. To address this gap in the literature, we investigated a role for E2 in modulating TGF-β1-induced signaling mechanisms and identified novel pathways impacted by estrogen in bronchial epithelial cells.

**Methods:**

We investigated a role for E2 in modulating TGF-β1-induced epithelial to mesenchymal transition (EMT) in bronchial epithelial cells (BEAS-2Bs) and characterized the effect of TGF-β1 on ESR mRNA and protein expression in BEAS-2Bs. We also quantified mRNA expression of ESRs in lung tissue from individuals with IPF and identified potential downstream targets of E2 signaling in BEAS-2Bs using RNA-Seq and gene set enrichment analysis.

**Results:**

E2 negligibly modulated TGF-β1-induced EMT; however, we report the novel observation that TGF-β1 repressed ESR expression, most notably estrogen receptor alpha (ESR1). Results of the RNA-Seq analysis showed that TGF-β1 and E2 inversely modulated the expression of several genes involved in processes such as extracellular matrix (ECM) turnover, airway smooth muscle cell contraction, and calcium flux regulation. We also report that E2 specifically modulated the expression of genes involved in chromatin remodeling pathways and that this regulation was absent in the presence of TGF-β1.

**Conclusions:**

Collectively, these results suggest that E2 influences unexplored pathways that may be relevant to pulmonary disease and highlights potential roles for E2 in the lung that may contribute to sex-specific differences.

**Electronic supplementary material:**

The online version of this article (10.1186/s12931-018-0861-5) contains supplementary material, which is available to authorized users.

## Background

Epidemiological studies have associated sex with idiopathic pulmonary fibrosis (IPF) where males are more negatively impacted and females have better survival rates [1–5]. In addition, a study by our group found a statistical interaction between sex and IPF severity based on gene expression in diseased lung tissue [6]. Based on these observations, several hypotheses suggest a role for estrogens and androgens.

Sex-based studies of pulmonary fibrosis in small animal models have produced mixed results. Several reports suggest that estrogen (E2) is protective and androgens exacerbate fibrotic responses. For example, bleomycin caused increased pulmonary fibrosis in male mice compared to females [7, 8], and a recent study found that it was the presence of the Y chromosome and not necessarily sex itself that predisposed the lung to increased bleomycin-induced fibrosis in male and female mice [9]. Conversely, a study in rats indicated that females exhibited increased pulmonary fibrosis in response to bleomycin compared to males [10]. Importantly, variable responses to bleomycin in mice may be due to differential activity of bleomycin hydrolase between male and females [11]. Conversely, E2 was protective in ovariectomized mice where a significant increase in total lung collagen content and airway subepithelial collagen deposition was observed in ovariectomized mice which was mitigated by E2 administration [12].

Current reports have not probed the molecular mechanisms impacted by hormones in the lung contributing to sex-based differences in fibrotic disease except one study which found increased TGF-β1 expression in rat fibroblasts exposed to E2 [10]. Regulation of TGF-β1 by E2 has been extensively characterized in other model systems and the effects appear to be contextual. For example, E2 inhibited TGF-β1 signaling in breast cancer cells by reducing the expression of activators of TGF-β1 [13] and by increasing degradation of SMAD proteins [14, 15]. Conversely, E2 increased TGF-β1 secretion in dermal fibroblasts [16], and TGF-β1 levels were reduced in the kidneys of diabetic female mice lacking estrogen receptor alpha (ESR1) compared to wildtype mice suggesting positive regulation of TGF-β1 by E2 [17].

E2 binds and activates several receptors including the nuclear transcription factors estrogen receptor alpha (ESR1), estrogen receptor beta (ESR2), and several variants thereof, and the putative membrane-bound G-protein coupled estrogen receptor 1 (GPER1). Studies aiming to decipher receptor-specific effects on TGF-β1 signaling have been limited to non-lung cells and tissues (i.e. breast). For example, Stope et al. found that ESR1 but not ESR2 inhibited TGF-β1 activation in breast cancer cells [18] while others found that both ESR1 and ESR2 suppressed TGF-β1 signaling by associating with and acting as a transcriptional corepressor for SMAD3 [19, 20]. Other studies showed a role for GPER1 in mediating E2-dependent reduction in SMAD protein activation in breast cancer cells [21] and TGF-β1-induced extracellular matrix (ECM) production in human and rat mesangial cells [22].

Given the preponderance of opposing actions of E2 on TGF-β1 in in vitro systems other than the lung and the epidemiological evidence suggesting a male sex-bias in IPF, we hypothesized that E2 would inhibit TGF-β1-induced signaling in lung epithelial cells. To test this hypothesis, we investigated the impact of E2 on TGF-β1-induced epithelial to mesenchymal transition (EMT), characterized the expression of ESRs in bronchial epithelial cells and lung tissue from individuals with IPF, and performed RNA-Seq analysis to identify targets of E2 in bronchial epithelial cells.

## Methods

### Chemicals

Recombinant human transforming growth factor beta1 (TGF-β1) was purchased from R&D Systems (> 97% purity, Catalog No. 240-B002, Minneapolis, MN) and 17β-Estradiol (E2) was purchased from Sigma (≥98% purity, Catalog No. E2758, Saint Louis, MO).

### Human lung samples

The human lung tissue samples used in this study were a kind gift from Dr. Andrew Bryant. The explanted lung tissue was obtained from subjects undergoing lung transplant for IPF and from lungs rejected for transplant from normal controls per the National Institutes of Health Lung Tissue Research Consortium (protocol no. 14-99-0011). This study consisted of fifteen patients, eight with IPF (*n* = 8) and seven controls (*n* = 7) described in detail in Table 1. A diagnosis of IPF was determined based on ATS criteria [23, 24]. The protocol for collection of lung tissue samples and subsequent studies were approved by the institutional review board at Vanderbilt University and the University of Florida [25]. Immediately after lung biopsy or resection, a portion of the lung was removed and flash-frozen in liquid nitrogen or dry ice and stored at − 80 °C.

**Table 1:** Patient demographic data

Characteristics	Controls	Patients
Male/Female	2/3	8/0
Age^a^	64 ± 14.78	61.5 ± 7.11
Smoking Status		
Never	4	6
Ever	1	2
FVC, % predicted^a^	105.2 ± 7.79	42.9 ± 14.39

^a^Mean ± SD

### Cell culture

Human bronchial epithelial cells (BEAS-2Bs, CRL-9609™) were purchased from ATCC and cultured according to manufacturer’s specifications. STR analysis and mycoplasma contamination testing was not performed. Cells were cultured in bronchial epithelial growth medium (BEGM) consisting of bronchial epithelial basal medium (BEBM, Lonza CC-3171, Walkersville, MD) and the BEGM SingleQuot Kit Supplements & Growth Factors (Lonza CC-4175) but exchanging gentamicin for Penicillin-Streptomycin-Neomycin (PSN) Antibiotic Mixture (Gibco 15,640, ThermoFisher Scientific Inc., Waltham, MA). Cells were cultured in T75 Corning™ U-Shaped Cell Culture Flasks (Corning 430,641 U, Fisher Scientific Co LLC, Pittsburgh, PA) coated with a matrix (4.5 mL per 75 cm^2^) consisting of 0.01 mg/mL fibronectin (Akron AK8350, City, State), 0.03 mg/mL bovine collagen (Gibco A10644–01), and 0.01 mg/mL BSA (Fisher BP1605) in BEBM. Cells were sub-cultured up to 11 times before use in exposure studies. All exposures were performed in BEGM without the supplied bovine pituitary extract (BPE) aliquot because its composition is not defined. For the gene expression experiments, BEAS-2Bs were plated at 40,000 cells/mL on matrix-coated 12-well Nunc™ Cell-Culture-Treated Multidishes (ThermoFisher Scientific Inc.), allowed to adhere overnight, and subsequently exposed for 48 h to the indicated concentrations of TGF-β1 dissolved in 0.1% BSA (Fisher BP1605) and 4 mM HCl or 10 nM E2 dissolved in DMSO (Mediatech Inc. MT25950CQC). Doses of TGF-β1 and E2 were chosen based on efficacy in previous experiments [26–30]. For the protein expression experiments, BEAS-2Bs were plated at 60,000 cells/mL on matrix-coated 6-well Corning® Costar® cell culture plates (Costar 3516), allowed to adhere overnight, and subsequently exposed for 48 h to the indicated concentrations of TGF-β1 in BEBM without BPE. All chemical solvent concentrations were maintained below 0.1%.

### Total RNA extraction and purification

Frozen whole lung samples were pulverized over liquid nitrogen using a mortar and pestle then mechanically disrupted in RNA STAT-60™ Reagent (Tel-Test, Inc. Cs-502, Friendswood, TX) using a handheld homogenizer, and cell lysates were collected in RNA STAT-60™ Reagent and vortexed to promote lysis. RNA was extracted per manufacturer’s specifications followed by overnight precipitation at − 20 °C using 100% molecular biology grade isopropanol (Fisher BioReagents™ BP26184) containing 0.067% *v*/v GlycoBlue™ Coprecipitant (Ambion® AM9515). Precipitated RNA was pelleted by centrifugation at 14000 x g for 45 min at 4 °C and purified by washing 2X with 75% molecular biology grade absolute ethanol (Fisher BioReagents™ BP28184). RNA pellets were reconstituted in 15 μL RNAsecure™ (Ambion® AM7010). RNA was quantified using a Synergy™ H1 plate reader (BioTek Instrument, Inc., Winooski, VT) and RNA integrity was analyzed using a Bioanalyzer 2100 instrument (Agilent Technologies, Santa Clara, CA).

### qPCR

RNA (1–2 μg) was DNase-treated using the PerfeCTa DNase I Kit (Quanta BioSciences 95,150-01 k, VWR International LLC, Suwanee, GA) and subsequently reverse transcribed using the qScript™ cDNA Synthesis Kit (Quanta BioSciences 95,047). cDNA was diluted 1:20 in RNase-DNase free water. Each 10 μL qPCR reaction contained 1× SsoAdvanced™ Universal SYBR® Green Supermix (Bio-Rad 172–5270, Hercules, CA), 850 nM forward and reverse primers, and 3.3 μL of the cDNA dilution. Per MIQE guidelines [31], gene specific primers and cycling parameters are displayed in Table 2. *ESR2* primers were purchased from Bio-Rad (Unique Assay ID: qHsaCID0013184). Each qPCR reaction was followed by melt curve analysis to verify primer specificity. Cq values were determined by regression method using the CFX Manager 2.1 software and quantified using the relative ΔΔCq method [32] or the ratio method [33] when indicated. In the case of no amplification, a Cq value of 40 was applied. For the cell culture experiments, target gene expression was normalized to glyceraldehyde 3-phosphate dehydrogenase (*GAPDH*) expression and to ribosomal protein S13 (*RPS13*) expression in the human lung tissue samples.

**Table 2:** Primer information for qPCR

Gene	Forward (5′-3′)	Reverse (5′-3′)	Protocol	Efficiency	Source
*GAPDH*	GAAGGTGAAGGTCGGAGTC	GAAGATGGTGATGGGATTTC	95C 3m; 95C 10s, 60C 30s, ×40	93.9%	[92]
*ESR1*	CCACCAACCAGTGCACCATT	GGTCTTTTCGTATCCCACCTTTC	95C 3m; 95C 10s, 60C 30s, ×40	100.7%	[93]
*ESR2*	Proprietary	Proprietary	95C 3m; 95C 10s, 60C 30s, ×40	102.9%	Bio-Rad
*GPER*	GCTCCCTGCAAGCAGTCTTT	GAAGGTCTCCCCGAGAAAGC	95C 3m; 95C 10s, 60C 30s, ×40	97.2%	[94]
*SNAI1*	CCAGACCCACTCAGATGTCAA	GGACTCTTGGTGCTTGTGGA	95C 3m; 95C 10s, 58C 30s, ×40	97.2%	[94]
*CDH1*	GAAAGCGGCTGATACTGACC	CTCAGACTAGCAGCTTCGGA	95C 3m; 95C 10s, 58C 30s, ×40	104.9%	[94]
*ACTA2*	CATCATGCGTCTGGATCTGG	GGACAATCTCACGCTCAGCA	95C 3m; 95C 10s, 60C 30s, ×40	94.8%	[95]
*MMP2*	TGTGTTCTTTGCAGGGAATG	TCCAGAATTTGTCTCCAGCA	95C 3m; 95C 10s, 58C 30s, ×40	93.6%	[96]
*CTGF*	AATGCTGCGAGGAGTGGGT	CGGCTCTAATCATAGTTGGGTCT	95C 3m; 95C 10s, 60C 30s, ×40	96.4%	[97]
*VIM*	GCGTGAAATGGAAGAGAACT	GGTATCAACCAGAGGGAGTG	95C 3m; 95C 10s, 56C 10s, 72C 30S, ×40	104.0%	[30]
*MUC15*	CCATCGGCGACTTTATGACG	TCTTCACTTTCTGGCATGGCT	95C 3m; 95C 10s, 60C 30s, ×40	92.1%	[94]

### Protein extraction and purification

After exposure, media was removed, and cells were washed 3X with ice-cold PBS and subsequently collected in 200 μL RIPA Lysis and Extraction Buffer (Pierce Biotechnology 89,900) containing Pierce™ Protease Inhibitor Tablets (Pierce Biotechnology 88,265) using a cell scraper. The lysates were passed 5X through a 25-gauge needle and incubated on ice for 30 min with intermittent mixing. Thereafter, the lysates were centrifuged at 14000 x g for 20 min at 4 °C, supernatants were removed, and total protein quantified using the Pierce BCA Protein Assay Kit (Pierce Biotechnology 23,225).

### Western blot

For SDS-PAGE, total protein (15 μg) was diluted in Novex™ Tris-Glycine SDS Sample Buffer (2X) (Invitrogen LC2676) and loaded onto a Novex™ WedgeWell™ 4–12% Tris-Glycine Mini Gel (Invitrogen XP04120BOX). Electrophoresis was performed for 30 min at 225 V and electrophoresed proteins were subsequently transferred to nitrocellulose membranes (GVS Life Sciences EP4HY00010) by semi-dry transfer method under 15 V for 30 min. Blots were blocked with 5% dehydrated milk dissolved in TBST for 1 h at room temperature, then incubated with mouse monoclonal antibody specific for estrogen receptor α (anti-ESR1, Santa Cruz Biotechnology, Inc. SC-514857, Dallas, TX) diluted 1:100 in blocking solution overnight at 4 °C. Blots were washed 3X in TBST for 10 min and incubated with HRP-linked Rabbit anti-Mouse IgG (H + L) Secondary Antibody (Pierce 31,450, Rockford, IL) diluted 1: 4000 in TBST for 1 h at room temperature. Blots were washed 3X with TBST for 10 min at room temperature, then 1X with TBS at room temperature. Thereafter, blots were incubated with 1:1 solution of Clarity™ Western ECL Blotting Substrates (Bio-Rad 170–5060, Hercules, CA) and imaged using the auto-exposure option on a Bio-Rad ChemiDoc™ MP system (Bio-Rad 17,001,402). After probing with anti-ESR1, blots were incubated 2X for 10 min at room temperature in mild stripping buffer (0.1 M Glycine, 20 mM MgAcetate, 50 mM KCl, pH 2.2), then washed 3X with TBST for 5 min at room temperature. To verify antibody stripping, the blot was probed with HRP-linked secondary antibody and re-imaged as before. After verification of anti-ESR1 removal, the blot was incubated in blocking solution for 1 h at room temperature, then incubated with mouse monoclonal antibody specific for β-Actin (anti-β-Actin, Sigma A5441) diluted 1:5000 in blocking solution overnight at 4 °C. The blot was subsequently washed as before, incubated in HRP-linked Rabbit anti-Mouse IgG (H + L) Secondary Antibody (Pierce 31,450) diluted 1:5000 in TBST for 1 h at room temperature, and imaged as before. Densitometry was performed in ImageJ [34] using the Gel Analysis method outlined in the ImageJ documentation.

### RNA-Seq library preparation and sequencing

RNA library construction was performed at the Interdisciplinary Center for Biotechnology Research (ICBR) Gene Expression Core, University of Florida (UF). RNA concentration was determined on Qubit® 2.0 Fluorometer (ThermoFisher/Invitrogen, Grand Island, NY), RNA quality was assessed using the Agilent 2100 Bioanalyzer. Total RNA with 28S/18S > 1 and RNA integrity number (RIN) ≥ 7 was used for RNA-seq library construction. The RINs of all total RNA samples were 9.7–10. 2 μL of 1:2000 diluted External RNA Controls Consortium (ERCC) Spike-In Mix (Ambion™ 4,456,740, ThermoFisher Scientific Inc.) was added to 100 ng of high-quality total RNA followed by mRNA isolation using NEBNext Poly(A) mRNA Magnetic Isolation module (New England Biolabs E7490, Ipswich, MA) and RNA library construction with NEBNext Ultra RNA Library Prep Kit for Illumina (New England Biolabs E7530) according to the manufacturer’s user guide. Specifically, 100 ng of total RNA together with 2 μL of 1:2000 diluted ERCC was added to extracted mRNA with 15 μL of NEBNext Magnetic Oligo d(T)25 and fragmented in NEBNext First Strand Synthesis Buffer by heating at 94 °C for 8 min, followed by first-strand cDNA synthesis using reverse transcriptase and random primers. Synthesis of ds cDNA was done using the 2nd strand master mix provided in the kit. The resulting double-stranded cDNA was end-repaired, dA-tailed and ligated with NEBNext adaptors. Finally, library was enriched by 13 cycles of amplification, and purified by Meg-Bind RxnPure Plus beads (Omega Biotek M1386, Norcross, GA). Bar-coded libraries were sized on the bioanalyzer, quantitated by QUBIT and qPCR (Kapa Biosystems KK4824, Wilmington, MA). Seventeen individual libraries were pooled at equal molar (20 nM).

Bar-coded cDNA was sequenced using the 2 × 100 configuration in 2 lanes of a HiSeq 3000 instrument (Illumina, San Diego, CA). The yield for the run was in the expected range, the quality was good with Q30 > 96.25%, and the pool was well-balanced (in terms of number of reads per samples).

### Bioinformatics

Short reads were trimmed and filtered to remove low-quality reads using Trimmomatic version 0.36. Quality control was assessed using the FastQC tool, version 0.11.4. Short reads were aligned to the transcriptome using STAR version 2.5.2a. Transcript quantification and differential analysis was performed using RSEM version 1.2.31. Differential analysis was performed at the level of coding genes, all transcript, and all splicing isoforms. Coding genes, transcripts, and splicing isoforms were considered statistically significant if FDR-corrected *p*-value ≤5% and fold change > 1.5 in either direction. Clustering analysis was performed using the ‘gplots’ package in R version 3.3.2 (2016-10-31). Gene set enrichment analysis was performed using Pathway Studio® Version 11.4.0.8 operating on the ResNet Mammalian database (Elsevier). Statistically significant enrichment (*p* ≤ 0.05) of predefined gene sets was determined by Mann-Whitney U-test. The data discussed in this publication have been deposited in NCBI’s Gene Expression Omnibus [35] and are accessible through GEO Series accession number GSE100574 (https://www.ncbi.nlm.nih.gov/geo/query/acc.cgi?acc=GSE100574).

### Statistics

Normality of experimental data was determined by D’Agostino & Pearson omnibus normality test, Shapiro-Wilk normality test, or KS normality test using GraphPad Prism software (Version 5.01, GraphPad Software, Inc.). Data were determined to be normal by passing at least one normality test (*p* < 0.05). For qPCR and western blot analyses, statistically significant differences (*p* < 0.05) in mean fold changes between experimental groups were determined by one-way ANOVA followed by Newman-Keuls multiple comparison test or unpaired, two-tailed t test when indicated using GraphPad Prism software. If the data were not normal, statistically significant differences were determined by two-tailed Mann-Whitney U test.

## Results

### TGF-β1 induces changes consistent with EMT

**Fig. 1:**
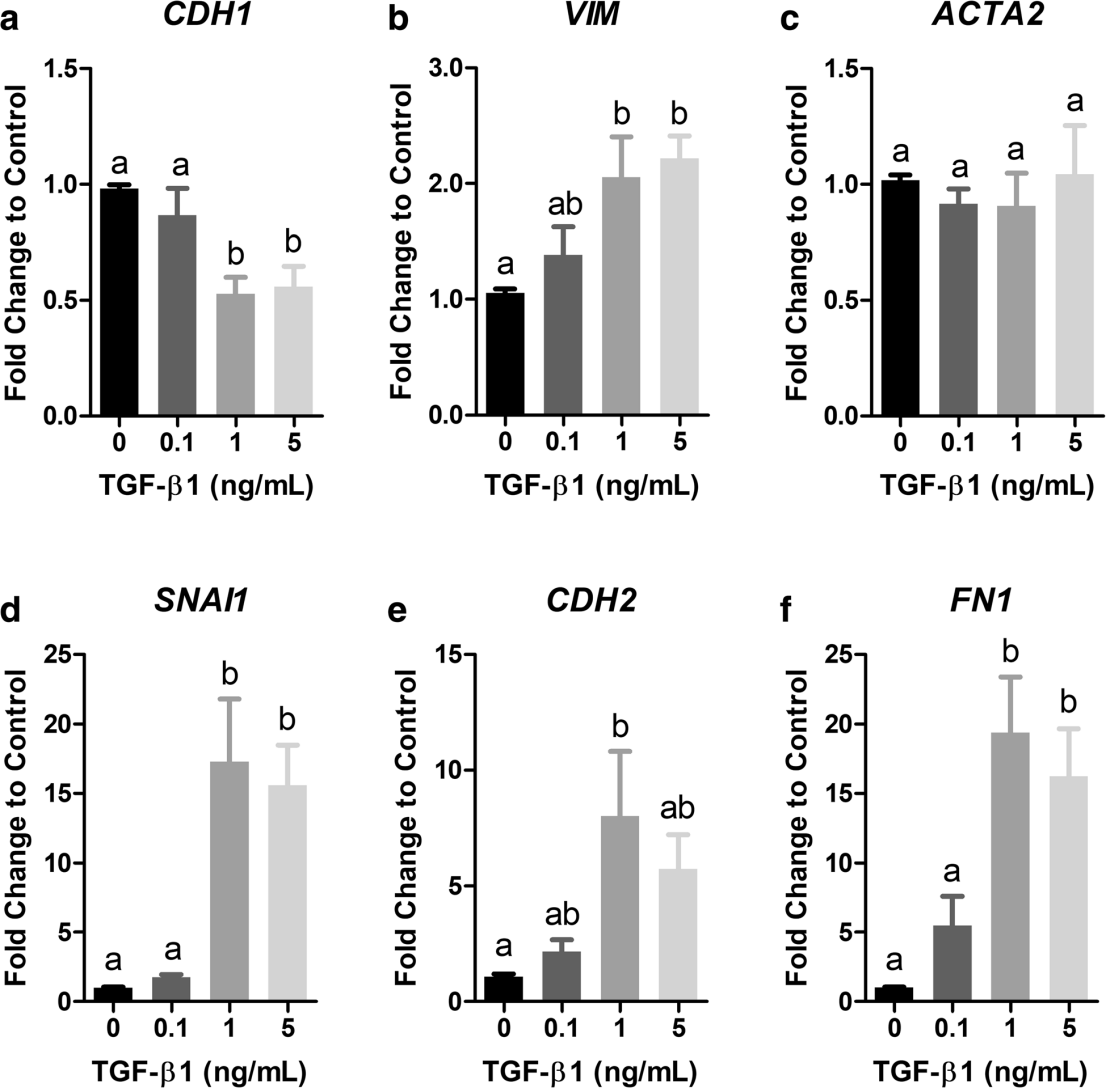
TGF-β1 induces EMT-like changes in mRNA expression in BEAS-2B cells. (**a-f**) BEAS-2B cells were exposed to TGF-β1 (0.1, 1, and 5 ng/mL) for 48 h and mRNA expression of the epithelial cell type marker, E-cadherin (*CDH1,* **a**), the mesenchymal cell type markers Vimentin (*VIM*, **b**), Snail family transcriptional repressor 1 (*SNAI1*, **d**), Cadherin 2 (*CDH2*, **e**), and Fibronectin (*FN1*, **f**), and the myofibroblast cell type marker, Alpha smooth muscle actin (*ACTA2*) was measured by qPCR. Target gene expression was normalized to *GAPDH* mRNA expression and quantified as fold change to control using the relative ΔΔCq method. Data are mean ± SEM of three or four independent experiments. Different letters indicate statistically significant (*p* < 0.05) differences between groups as determined by one-way ANOVA and Newman-Keuls multiple comparison test

To begin investigations, we optimized a well-characterized model of TGF-β1-induced EMT based on changes in gene expression. BEAS-2Bs were exposed to TGF-β1 (0.1, 1, and 5 ng/mL) for 48 h and mRNA expression of molecular markers for EMT were assayed by qPCR. Results revealed a dose-dependent response where exposure of cells for 48 h to 1 and 5 ng/mL TGF-β1 caused a significant reduction in expression of the epithelial cell type marker, E-cadherin (*CDH1*), compared to control cells (Fig. 1a). Conversely, exposure of cells to 1 and 5 ng/mL TGF-β1 caused a significant increase in expression of the mesenchymal cell type markers, Vimentin (*VIM*), Snail family transcriptional repressor 1 (*SNAI1*), N-cadherin (*CDH2*), and Fibronectin (*FN1*) compared to control cells (Fig. 1b, d-f). Exposure of cells to TGF-β1 did not affect expression of the myofibroblast cell type marker, alpha smooth muscle actin (*ACTA2*) at any tested doses (Fig. 1c).

### E2 does not significantly affect TGF-β1-induced EMT

**Fig. 2:**
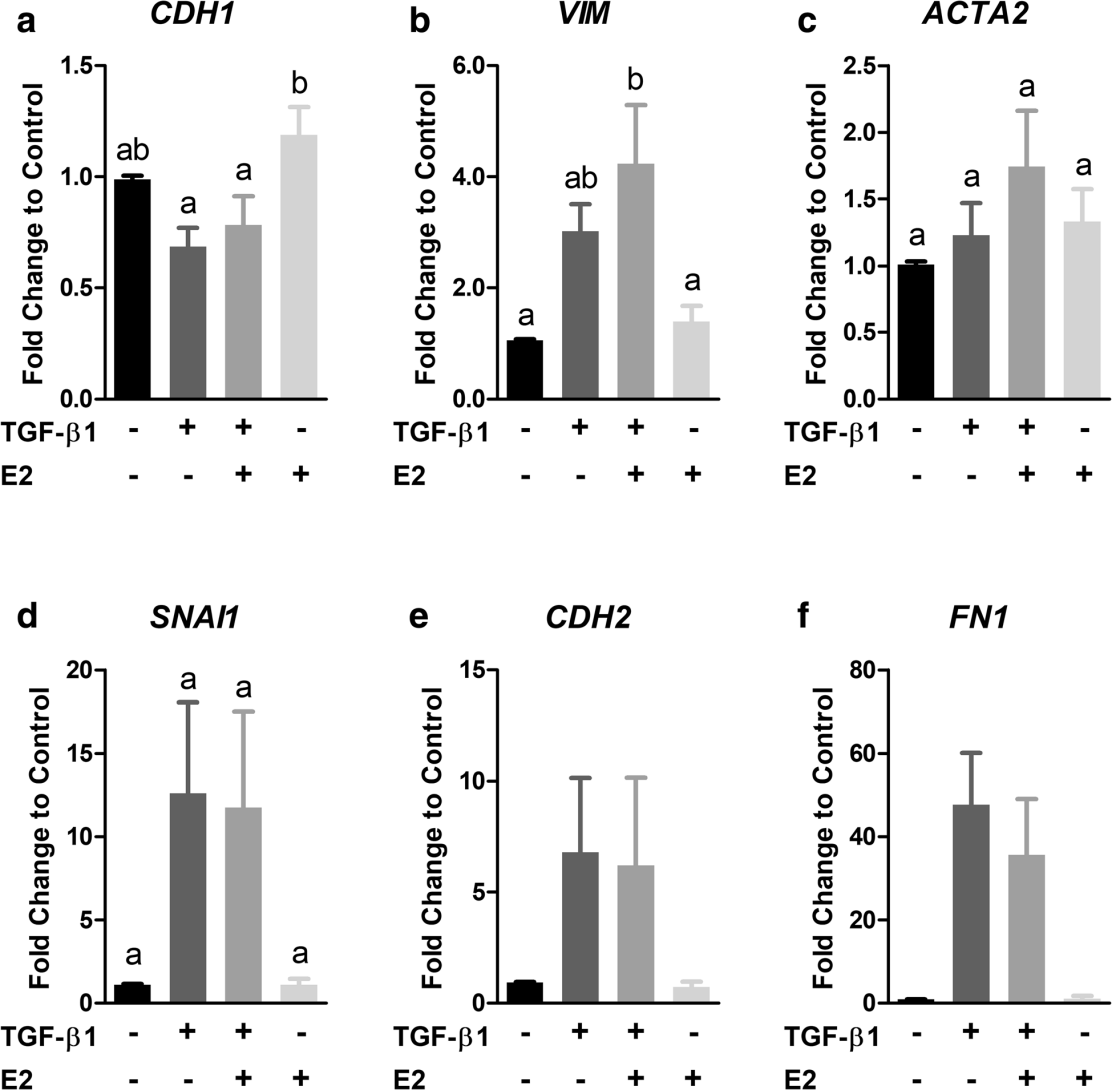
E2 does not significantly affect TGF-β1-induced EMT. BEAS-2B cells were exposed to 5 ng/mL TGF-β1 in the presence or absence of 10 nM E2 for 48 h. (**a-f**) Expression of *CDH1* (**a**), *VIM* (**b**), *ACTA2* (**c**), *SNAI1* (**d**), *CDH2* (**e**), and *FN1* (**f**) mRNA was measured by qPCR. Target gene expression was normalized to *GAPDH* mRNA expression and quantified as fold change to control using the relative ΔΔCq method. Data are mean ± SEM of three or four (**a-d**) or two (**e-f**) independent experiments. Different letters indicate statistically significant (*p* < 0.05) differences between groups as determined by one-way ANOVA and Newman-Keuls multiple comparison test

A role for E2 in modulating TGF-β1-induced EMT was assessed by exposing BEAS-2B cells to 5 ng/mL TGF-β1 in the presence and absence of 10 nM E2 for 48 h. Thereafter, expression of EMT marker genes was assayed by qPCR. As expected, 5 ng/mL TGF-β1 caused a reduction (0.686-fold, *p* > 0.05) in *CDH1* mRNA expression (Fig. 2a). However, the addition of 10 nM E2 did not significantly affect the TGF-β1-induced response or *CDH1* expression individually (Fig. 2a). Exposure of cells to 5 ng/mL TGF-β1 caused a trend of increased mRNA expression of the mesenchymal markers, *VIM* (*p* > 0.05), *SNAI1* (*p* > 0.05), *CDH2*, *FN1* (Fig. 2b, d-f) and similar to *CDH1*, exposure of cells to 5 ng/mL TGF-β1 in the presence of 10 nM E2 did not result in a statistically significant difference from cells exposed to TGF-β1 individually (Fig. 2b, d-f). As before, there were no statistically significant differences in expression of *ACTA2* compared to control cells in any exposure group, however there was a 1.74-fold trend of increased expression in the co-exposure group (Fig. 2c). Statistically significant differences could not be determined between groups for *CDH2* and *FN1* because these data were generated from only two independent experiments.

### TGF-β1 reduces estrogen receptor mRNA and protein expression

**Fig. 3:**
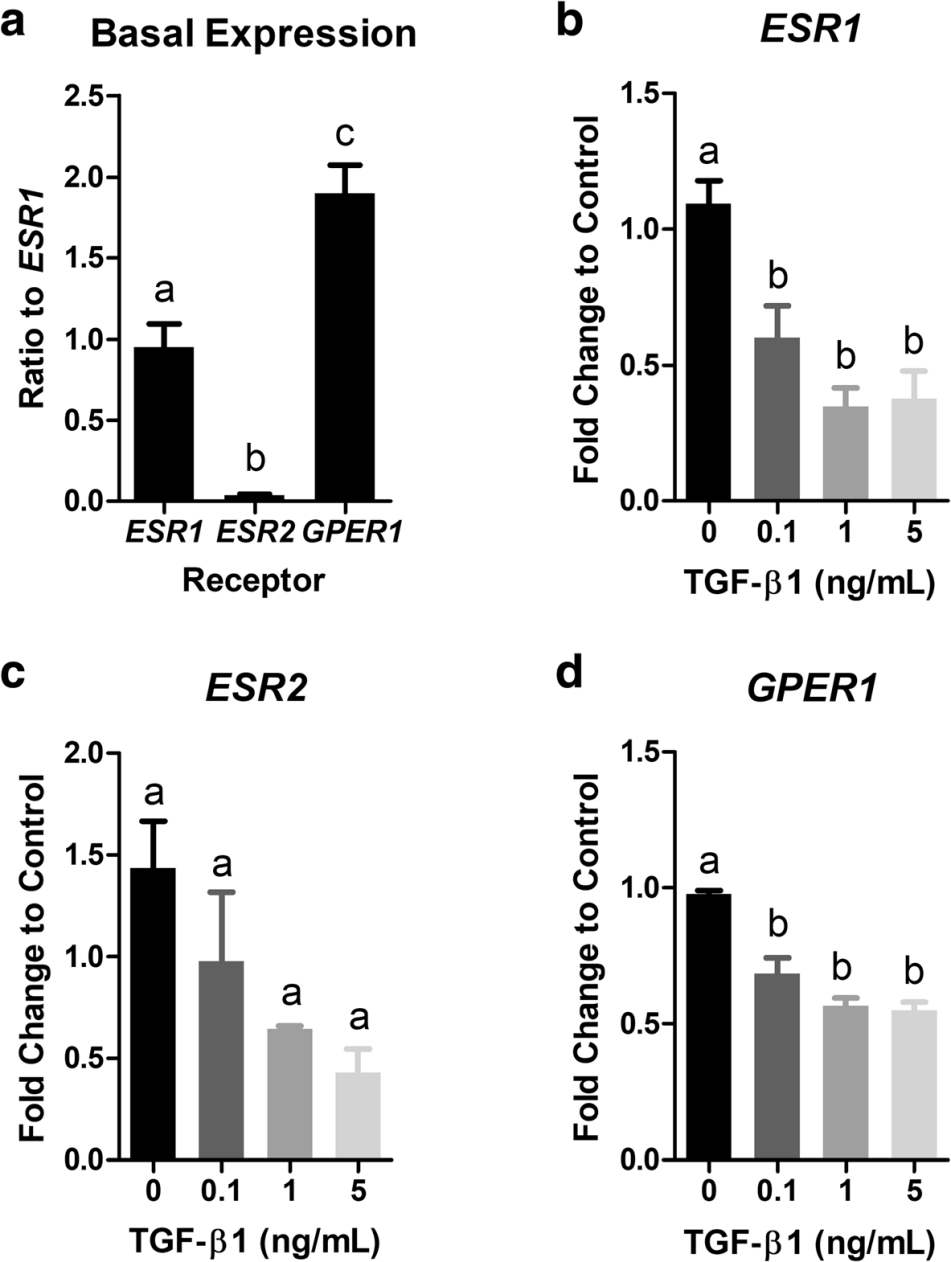
TGF-β1 down-regulates *ESR1*, *ESR2*, and *GPER1* mRNA expression in BEAS-2Bs. (**a**) Relative expression of estrogen receptor subtypes in control cells was *GPER1* > *ESR1* > *ESR2*. ESR gene expression was normalized to *GAPDH* mRNA expression and calculated as a ratio to *ESR1* mRNA expression. (**b-d**) BEAS-2B cells were exposed to TGF-β1 (0.1, 1, and 5 ng/mL) for 48 h and expression of *ESR1* (*n* = 3; **b**), *ESR2* (*n* = 2; **c**), and *GPER1* (*n* = 3; **d**) mRNA was measured by qPCR. Target gene expression was normalized to *GAPDH* mRNA expression and quantified as fold change to control using the relative ΔΔCq method. Data are mean ± SEM and different letters indicate statistically significant (*p* < 0.05) differences between groups as determined by one-way ANOVA and Newman-Keuls multiple comparison test

Next, we investigated whether estrogen receptor mRNA expression was was a target for TGF-β1. The baseline expression levels of estrogen receptor alpha (*ESR1*), estrogen receptor beta (*ESR2*), and g-protein coupled estrogen receptor (*GPER1*) in control cells were expressed as a ratio to *ESR1* based on the method described by Pfaffl et al. [33] to determine relative baseline expression levels. The relative expression of each receptor subtype was *GPER1* > *ESR1* > *ESR2* (Fig. 3a). Exposing BEAS-2Bs to increasing concentrations of TGF-β1 (0.1, 1, and 5 ng/mL) for 48 h caused a 1.81-, 3.11-, and 2.87-fold (*p* < 0.05) decrease in *ESR1* mRNA expression compared to controls (Fig. 3b). Similar trends were observed for *ESR2* mRNA expression compared to controls (Fig. 3c), and a 1.44, 1.72, and 1.78-fold (*p* < 0.05) decrease in *GPER1* mRNA expression compared to controls (Fig. 3d) was observed for the three doses, respectively.

**Fig. 4:**
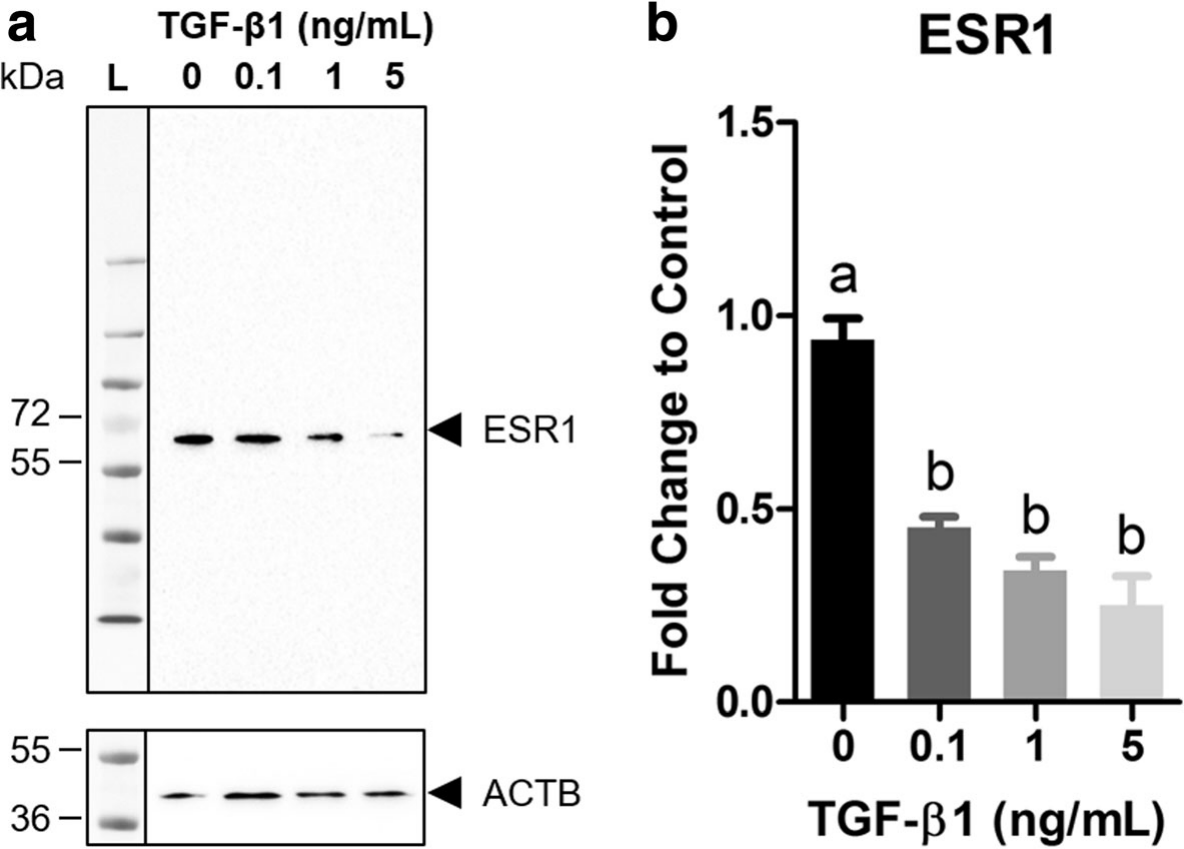
TGF-β1 down-regulates ESR1 protein expression. BEAS-2B cells were exposed to TGF-β1 (0.1, 1, and 5 ng/mL) for 48 h and ESR1 protein expression was measured by western blot followed by densitometric analysis in ImageJ. **a** Representative western blot. **b** Fold change ESR1 protein expression was normalized to Beta-actin (ACTB) and calculated as fold change to vehicle control (0 ng/mL TGF-β1). Data are mean ± SEM normalized arbitrary density units of duplicate measurements per blot of three independent experiments. Different letters indicate statistically significant (*p* < 0.05) differences between groups as determined by one-way ANOVA and Newman-Keuls multiple comparison test

BEAS-2Bs were exposed to TGF-β1 (0.1, 1, and 5 ng/mL) for 48 h and ESR1 was detected by western blot to determine if TGF-β1 reduced ESR1 protein levels (Fig. 4a). Signal intensity was quantified by densitometry using ImageJ (Fig. 4b). Similar to the mRNA results, TGF-β1 (0.1, 1, and 5 ng/mL) caused a 2.09-, 2.77-, and 3.76-fold significant decrease in ESR1 protein levels, respectively (Fig. 4b).

### Estrogen receptor mRNA expression is reduced in lungs of patients with IPF

**Fig. 5:**
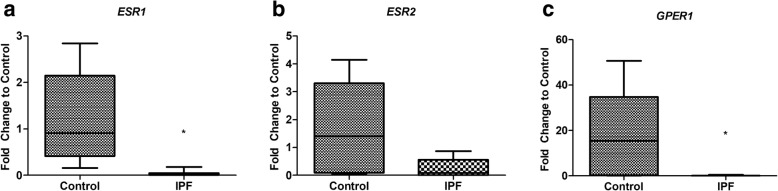
Estrogen receptor mRNA expression is reduced in lungs of patients with severe IPF compared to healthy control subjects. **a-c** Gene expression of *ESR1* (**a**), *ESR2* (**b**), and *GPER1* (**c**) was measured in lung tissue from patients with IPF and healthy controls by qPCR. Target gene expression was normalized to *GAPDH* mRNA expression and quantified as fold change to control using the relative ΔΔCq method. Box plots represent 5–95% confidence intervals and asterisks (*) indicate statistically significant (*p* < 0.05) differences compared to controls as determined by two-tailed Mann-Whitney U test

We next questioned whether our in vitro results translated in vivo. To answer this, mRNA expression of *ESR1*, *ESR2*, and *GPER1* was compared in lung tissue from healthy controls to individuals with end-stage IPF given that those with IPF tend to have higher TGF-β1 serum levels compared to healthy controls [36]. A qPCR analysis found that *ESR1* and *GPER1* mRNA expression was significantly reduced in the lungs of patients with end-stage IPF compared to healthy controls while there was a trend of reduced expression of *ESR2* in the former group (Fig. 5a-c).

### TGF-β1 and E2 exhibit unique transcriptional profiles

**Fig. 6:**
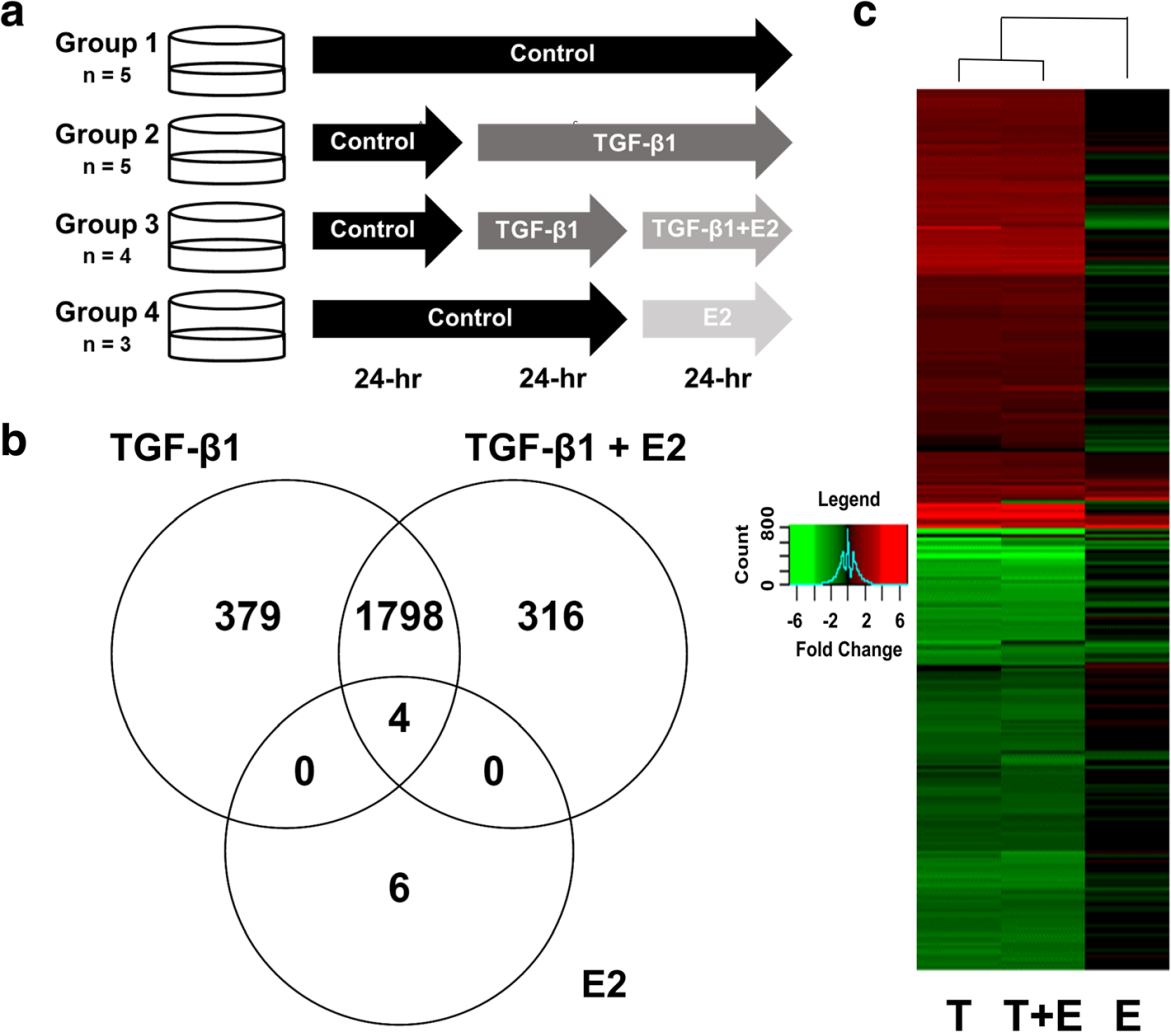
TGF-β1 and E2 exhibit distinct transcriptional profiles. **a** BEAS-2Bs were exposed to 5 ng/mL TGF-β1 and 10 nM E2 individually and in combination. Cells were acclimated for 24 h, then groups 2 and 3 were exposed to TGF-β1. After 24 h, groups 3 and 4 were exposed to E2, and all samples were collected 24 h thereafter. **b** Venn diagram highlighting distribution of differentially expressed genes [Log2(Fold Change) ≥ |0.6| and FDR-corrected *p*-value < 0.05] among the treatment groups. **c** Heat map showing the clustering and relative expression levels [Log2(Fold Change) compared to controls] of genes that were differentially expressed in at least one treatment group. Red coloring indicates up-regulation compared to controls and green coloring indicates down-regulation compared to controls, (T, TGF-β1; T + E, TGF-β1 + E2; E, E2)

**Fig. 7:**
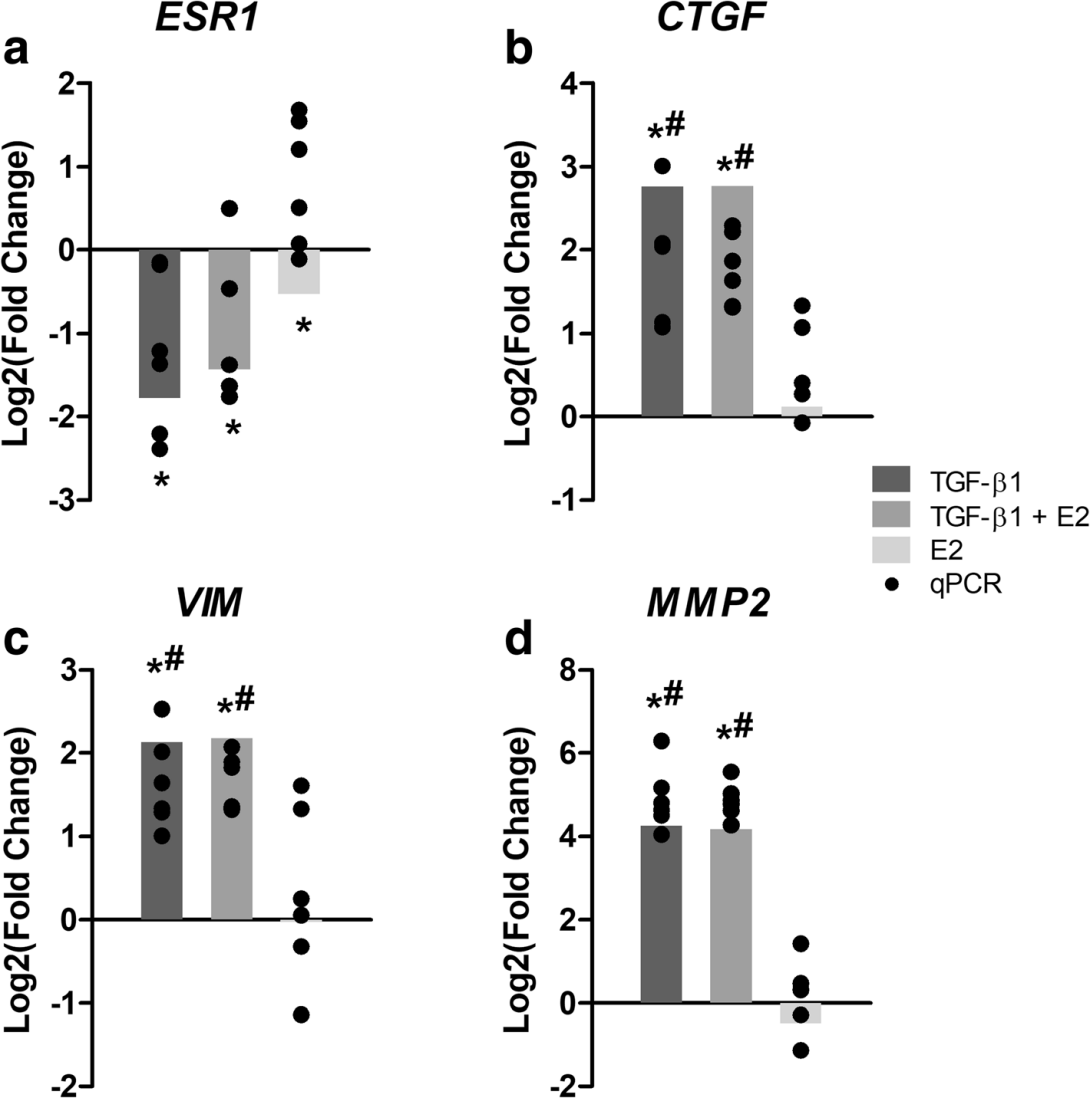
Orthogonal validation of RNA-Seq data. **a-d** Expression of select genes was validated by qPCR; *ESR1* (**a**), Connective tissue growth factor (*CTGF,* **b**), *VIM* (**c**), and Matrix metalloproteinase 2 (*MMP2*, **d**), in an identical and independent experiment. Bars represent expression [Log2(Fold Change)] of each gene in the RNA-Seq analysis, and black dots represent expression [Log2(Fold Change)] in each sample (*n* = 6) in the orthogonal experiment as determined by qPCR relative to vehicle control (DMSO). Target gene expression as measured by qPCR was normalized to *GAPDH* mRNA expression and quantified as fold change to control using the relative ΔΔCq method. Asterisks (*) indicate differential expression compared to controls [Log2(Fold Change) ≥ |0.6| and FDR-corrected *p*-value < 0.05] in the RNA-Seq analysis, and pound signs (#) indicate statistically significant (*p* < 0.05) differences compared to vehicle controls in the qPCR data as determined by one-way ANOVA and Newman-Keuls multiple comparison test

RNA-Seq was performed to identify transcriptional targets of E2 in bronchial epithelial cells and cellular processes that may be affected by the observed down-regulation of ESR expression by TGF-β1. For this experiment, BEAS-2Bs were exposed to either vehicle control, 5 ng/mL TGF-β1 for 48 h, 10 nM E2 for 24 h, or pre-exposed to 5 ng/mL TGF-β1 for 24 h and subsequently co-exposed to both 5 ng/mL TGF-β1 and 10 nM E2 for 24 h (Fig. 6a). Differential expression analysis resulted in 2182 coding genes with FDR-corrected *p*-value ≤0.05 and Log2(Fold Change) > |0.6| compared to controls in the TGF-β1 group. The expression of 2119 coding genes was altered in the group co-exposed to TGF-β1 and E2, and 10 in the group exposed to E2. In sum, 379, 316, and 6 genes were specifically altered in the TGF-β1, TGF-β1 + E2, and E2 groups, respectively, while 1798 genes were differentially regulated in both the TGF-β1 and TGF-β1 + E2 groups, and 4 were differentially regulated in all groups (Fig. 6b). Many of the genes significantly up-regulated by TGF-β1, such as CTGF, MMP2 and VIM, are well-known targets of this pathway (Fig. 7, Additional file 1: Table S1). Other genes significantly up-regulated in all treatment groups included Sprouty RTK signaling antagonist 4 (*SPRY4*) and Dual specificity phosphatase 6 (*DUSP6*), and significantly down-regulated genes included Potassium voltage-gated channel subfamily Q member 1 (*KCNQ1*) and RAS protein activator like 1 (*RASAL1*) (Additional file 1: Tables S1-S2, Table 3). Genes that were specifically regulated by E2 included Retinol binding protein 7 [*RBP7*, Log2(Fold Change) = − 1.65] and Chloride intracellular channel 3 [*CLIC3,* Log2(Fold Change) = − 0.73] (Table 3). A hierarchical clustering analysis of genes differentially regulated in at least one exposure group showed that the expression profiles of the TGF-β1 and TGF-β1 + E2 group were more similar to each other than to the expression profile of E2 (Fig. 6c).

**Table 3:** Genes differentially regulated by E2

ENSEMBL Gene ID	Gene	Description	Log2(Fold Change)	*p*-value
ENSG00000258588	*TRIM6-TRIM34*	Tripartite Motif-Containing 6 And Tripartite Motif-Containing 34	5.36	1.33E-06
ENSG00000256966	*RP11-613 M10.8*	AL513165.2	3.61	2.98E-02
ENSG00000274944	*RP5-864 K19.6*	AL139260.3	2.93	1.34E-14
ENSG00000255439	*RP11-196G11.1*	AC135050.2	1.43	9.12E-03
ENSG00000187678	*SPRY4*	sprouty RTK signaling antagonist 4	0.76	3.20E-05
ENSG00000139318	*DUSP6*	dual specificity phosphatase 6	0.75	2.25E-08
ENSG00000169583	*CLIC3*	chloride intracellular channel 3	−0.73	1.40E-06
ENSG00000053918	*KCNQ1*	potassium voltage-gated channel subfamily Q member 1	−0.86	4.87E-03
ENSG00000111344	*RASAL1*	RAS protein activator like 1	−1.34	1.79E-03
ENSG00000162444	*RBP7*	retinol binding protein 7	−1.65	3.08E-02

The expression of genes relevant to pulmonary fibrosis and this current work was validated by qPCR in an independent experiment (Fig. 7). As expected, exposure to TGF-β1 caused a significant reduction in *ESR1* mRNA expression (Fig. 7a) and increased the expression of known targets of TGF-β1 such as Connective tissue growth factor (*CTGF,* Fig. 7b), *VIM* (Fig. 7c), and Matrix metalloproteinase 2 (*MMP2*, Fig. 7d). The presence of E2 in the co-exposure group did not have a clear effect on the expression of these genes compared to the TGF-β1 group.

### TGF-β1 and E2 differentially regulate gene sets

**Fig. 8:**
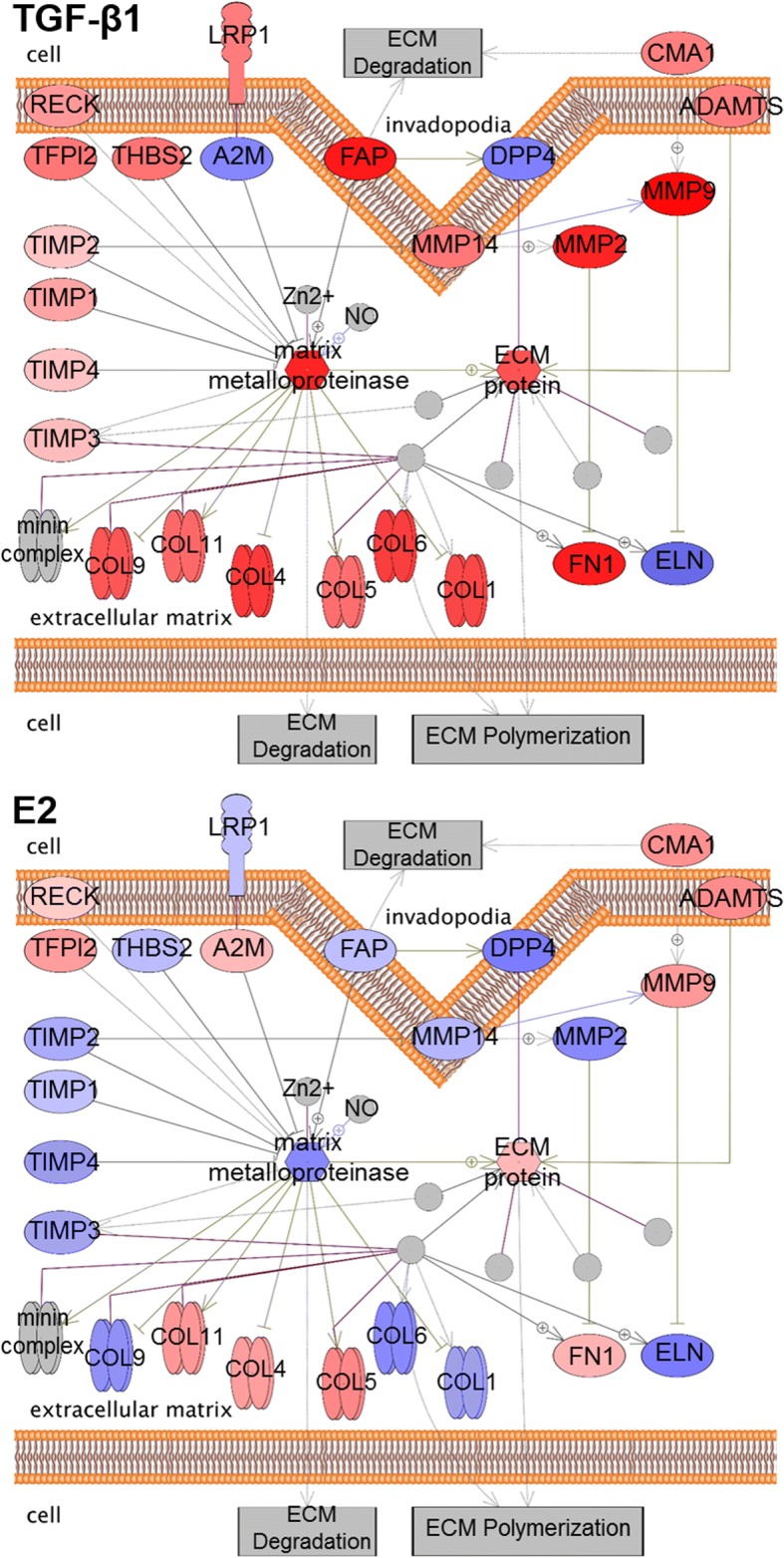
TGF-β1 and E2 cause differential regulation of genes involved in extracellular matrix turnover. A gene set enrichment analysis using Pathway Studio of genes identified by RNA-Seq revealed that exposure to 5 ng/mL TGF-β1 (top) and 10 nM E2 (bottom) caused statistically significant (*p* < 0.05) enrichment of the extracellular matrix turnover pathway. Gray boxes denote cellular processes involved in the extracellular matrix turnover pathway. Red proteins indicate up-regulation and blue proteins indicate down-regulation as determined by RNA-Seq

Gene set enrichment analysis (GSEA) [37] was performed to identify statistical enrichment in the RNA-Seq data of curated pathways using Pathway Studio® Version 11.4.0.8 (Elsevier). The GSEA resulted in differential enrichment of biological function and disease pathways among the exposure groups. As expected, exposure to TGF-β1 resulted in enrichment of pathways such as ECM turnover and skin fibrosis (Table 4). Exposure to TGF-β1 also resulted in statistical enrichment of pathways including alveolar epithelial cell dysfunction, Ca2+ flux regulation, classical and alternative complement pathways, and neutrophil chemotaxis (Table 4). In most cases, similar enrichment and median changes were observed in the co-exposure group (TGF-β1 + E2) and the TGF-β1 only exposure group except for alveolar epithelial cell dysfunction which was not statistically enriched in the co-exposure group (Table 4). E2 also caused enrichment of classical and alternative complement pathways, airway smooth muscle cell contraction, Ca2+ flux regulation, and ECM turnover, however, the median change of the latter two pathways was inverse (down-regulated) compared to the median change observed in the TGF-β1 and co-exposure groups (Table 4). The ECM pathway is presented graphically to highlight the inverse regulation of genes in the pathway by TGF-β1 (Fig. 8, top) and E2 (Fig. 8, bottom). E2 specifically caused statistical enrichment of pathways including histone acetylation and phosphorylation pathways, nucleosome-remodeling factor (NURF) in chromatin remodeling, and vasodilation activation (Table 4).

**Table 4:** Significantly enriched gene sets

Name		TGF-β1		TGF-β1 + E2		E2	
	Pathway Type	Median Change	*p*-value	Median Change	*p*-value	Median Change	*p*-value
Airway Smooth Muscle Cell Contraction	Diseases	1.14	4.72E-04	1.10	4.34E-03	−1.01	3.96E-02
Alveolar Epithelial Cell Dysfunction	Diseases	1.01	4.98E-02	–	–	–	–
Ca2+ Flux Regulation	Biological Function	1.02	2.12E-07	1.06	1.40E-05	−1.00	5.48E-09
Complement Alternative Pathway	Biological Function	−1.63	3.17E-04	−1.49	2.64E-05	−1.11	1.36E-03
Complement Classical Pathway	Biological Function	−1.34	2.27E-04	−1.49	3.09E-05	−1.15	4.87E-04
Extracellular Matrix Turnover	Biological Function	1.55	2.26E-08	1.53	3.98E-07	−1.04	6.86E-04
Histone Acetylation	Biological Function	–	–	–	–	1.04	3.68E-02
Histone Phosphorylation	Biological Function	–	–	–	–	1.05	2.99E-05
Neutrophil Chemotaxis	Biological Function	1.09	1.27E-03	1.07	7.25E-03	–	–
NURF in Chromatin Remodeling	Biological Function	–	–	–	–	1.06	2.48E-02
Skin Fibrosis	Diseases	1.07	2.15E-02	1.06	1.64E-02	–	–
Vasodilation Activation	Biological Function	–	–	–	–	−1.00	3.34E-02

(−), not significantly enriched in specified exposure group

## Discussion

This work was motivated by evidence suggesting hormones may influence gene regulation in the lung and contribute to sex differences in pulmonary diseases such as fibrosis. Using a well-established model lung epithelial cell line, we investigated the impact of E2 on TGF-β1-induced EMT. We report that although TGF-β1-induced EMT was not significantly affected by E2, this may be due to the novel observation that TGF-β1 repressed ESR expression, most notably ESR1. We extended this observation to identify novel targets of E2 by RNA-Seq that may be susceptible to TGF-β1-induced repression of ESRs such as chromatin remodeling processes and ECM turnover.

We first characterized the relative expression levels of the ESRs in our model cell line. We found that *GPER1* was the most abundant followed by *ESR1* while *ESR2* was least expressed (Fig. 3a). Our results are similar to a study by Stabile et al. that found higher expression of *ESR1* than *ESR2* in human lung adenocarcinomas and squamous cell lung tumors although a difference between *ESR1* and *ESR2* expression was not evident in normal lung cells [38]. These results are in contrast to a study by Mollerup et al. that found that *ESR2* was more abundantly expressed than *ESR1* [39] and another study by Couse et al. that found greater expression of *ESR2* in mouse lung [40]. The discrepancies may be a result of variable detection methods [38–40].

We probed a role for E2 in modulating TGF-β1-induced EMT because this process has been observed in fibrosis models although the relative contribution to fibrogenesis in humans is heavily debated [41–45]. TGF-β1 caused a significant reduction in expression of the epithelial cell type marker and a significant increase in expression of the mesenchymal cell type markers (Fig. 1b, d-f) similar to other studies [28–30]. Unlike the study by Doermer et al., we did not measure a significant increase in expression of *ACTA2* mRNA (Fig. 1c) which may be a result of the duration of exposure as this particular marker tends to be more highly induced at later time-points (5 days) [29] and is indicative of further differentiation of fibroblasts into contractile myofibroblasts [46].

Using this model system, we determined whether E2 affected TGF-β1-induced EMT. A role for E2 in inhibiting EMT in humans was suggested in a study which found that reduced expression of *ESR1* was associated with increased expression of genes involved in EMT in endometrial carcinoma samples [47]. Further, EMT is a target for sex hormones in cell types such as breast and prostate cancer cells where E2 signaling maintains an epithelial phenotype and suppresses EMT [48–51]. In our analysis, exposure to E2 did not significantly affect EMT marker gene expression individually nor did it impact the normal TGF-β1 response (Fig. 2a-f). Co-exposure to TGF-β1 and E2 resulted in a trend of increased expression of *VIM* and *ACTA2* compared to TGF-β1 alone which is consistent with one study that reported E2 promoted reversible EMT-like transition and collective motility in breast cancer cells [52].

E2 may not affect TGF-β1-induced EMT due to direct actions of TGF-β1 on ESRs themselves. Interestingly, we found that exposure to TGF-β1 caused a dose-dependent and significant reduction in *ESR1, ESR2*, and *GPER1* mRNA expression (Fig. 3b-c). We extended this to show that TGF-β1-induced repression of *ESR1* persisted at the protein level (Fig. 4a-b). Other studies have shown that TGF-β1 reduces *ESR1* mRNA expression [53, 54] and ESR1 protein expression [54, 55] in breast epithelial cancer cells and ESR2 protein expression in prostate cancer cells [51], however this is the first study to show that TGF-β1 reduced *ESR2* and *GPER1* mRNA expression and certainly the first to report any interaction between TGF-β1 and ESRs in bronchial epithelial cells. In support of this interaction occurring in vivo, we observed reduced expression of *ESR1*, *ESR2*, and *GPER1* in lung tissue from patients with end-stage IPF compared to healthy control subjects (Fig. 5a-c). Although we do not have measurements of TGF-β1 in our samples, others have shown increased serum TGF-β1 levels in patients with IPF compared to healthy controls [36]. These results should be carefully interpreted given the small sample size and the absence of mechanistic information linking increased serum TGF-β1 levels to *ESR1*, *ESR2*, and *GPER1* mRNA expression. Future studies should investigate which signaling mediators downstream of TGF-β1, e.g. SMADs, CTGF, or SNAI1, among others, are responsible for the observed repression.

Few studies to date have revealed a functional role for E2 in lung cells as measured by genes and pathways modulated downstream. Using RNA-Seq, we searched for enriched pathways in BEAS-2Bs exposed to E2 and TGF-β1 individually, and in combination, to both identify points of convergence of E2 and TGF-β1 signaling and to highlight novel E2 targets that may be susceptible to TGF-β1-induced repression of ESRs. Sequencing data indicated greater regulation of genes in response to TGF-β1 exposure in comparison to E2 exposure, perhaps consistent with the well-recognized strong pro-fibrotic response associated with TGF-β1. Although some genes were differentially regulated in the TGF-β1 or TGF-β1 + E2 exposure groups, most were shared suggesting that the presence of E2 had a minimal effect on the TGF-β1-induced transcriptome (Fig. 5b) potentially due to TGF-β1-induced repression of ESRs. We confirmed the expression of selected genes relevant to this work and/or known to be targets of TGF-β1 (Fig. 6, *ESR1, VIM, CTGF, MMP2*) which was consistent with our previous results and those reported in the literature [56–64].

Exposure to E2 did not induce as robust a transcriptional response in BEAS-2Bs compared to TGF-β1. However, we identified statistically significant regulation of 10 genes by E2 (Table 3) that have not been previously reported. Two genes that were specifically down-regulated by E2 included Chloride intracellular channel 3 (*CLIC3*) and Retinol binding protein 7 (*RBP7*) (Table 3). CLIC3 promotes migration and invasion of cancer cells by facilitating the functions of MT1-MMP (*MMP14*) [65, 66]. MT1-MMP is the most highly expressed MMP in IPF lungs [63] and may protect against PF by degrading collagen [67] and promoting lung repair [68]. Another study indicated that MT1-MMP promoted pulmonary fibrosis by activating latent TGF-β1 [69]. Our results suggest E2 may repress MT1-MMP function by downregulating *CLIC3* mRNA expression. RBP7, also known as CRABP4, is a retinol binding protein thought to play an important role in retinol uptake, storage, and metabolism [70]. *RBP7* has been shown to be up-regulated in IPF lung tissue [71] and in wound tissue in the normal chicken chorioallantoic wound model [72] although its role in fibrosis is unclear. *RBP7* is positively regulated by E2 in breast cancer cells [73] and mouse mammary gland [74]. The discrepancy in regulation in our study may be a result of variable exposure dynamics as the study by Calvo et al. exposed mice to one dose of E2 and sacrificed the animals 3 h later [74] while we exposed cells in vitro for 24 h. Nonetheless, E2 appears to regulate *RBP7* which may exhibit an unexplored effect on fibrogenic signaling.

As expected, exposure to TGF-β1 resulted in significant enrichment of the ECM turnover (Fig. 8a), alveolar epithelial cell dysfunction, and skin fibrosis pathways as determined by GSEA (Table 4). It is well known that TGF-β1 is involved in organization of the ECM [75] and neutrophil chemotaxis [76], and one of the prevailing hypotheses in IPF research is that it is a result of dysfunctional behavior of alveolar epithelial cells [77]. In this case, skin fibrosis serves as a surrogate for pulmonary fibrosis because the underlying mechanisms are similar and largely regulated by TGF-β1 [78], and pulmonary fibrosis does not exist as a curated, predefined pathway in Pathway Studio. In most cases, pathways enriched in the TGF-β1 individual exposure group were also enriched in the TGF-β1 + E2 co-exposure group, and the overall directionality as indicated by the median change was similar. This suggests that E2 has a limited effect on TGF-β1 once the pathways are in motion and/or was a result of TGF-β1-induced repression of ESRs thus mirroring the results seen at the gene level (Fig. 3b-c).

Interestingly, exposure to E2 resulted in specific enrichment of multiple pathways involved in epigenetic regulation of chromatin structure and organization including Histone Acetylation, Histone Phosphorylation, and NURF in Chromatin Remodeling (Table 4). While E2 has been shown to regulate histone acetylation in A549 cells [79], little is known about a role for E2 in transcriptionally regulating the expression of genes involved in chromatin remodeling in the lung. This is important because evidence for the importance of epigenetics and chromatin organization in lung disease is growing, particularly in the context of pulmonary fibrosis [80–85]. For example, histone deacetylases are involved in activation of lung fibroblasts to myofibroblasts [86] and accumulation of ECM components and EMT in the diabetic kidney [87]. Notably, exposure to TGF-β1 individually and in the presence of E2 did not result in enrichment of chromatin remodeling gene sets. This suggests that the absence of enrichment in the co-exposure group, despite the presence of E2, may be a result of TGF-β1-induced repression of ESR expression and not through direct regulation of genes by TGF-β1.

Similar to TGF-β1, exposure to E2 resulted in statistical enrichment of genes associated with ECM turnover, airway smooth muscle cell contraction, and calcium flux regulation pathways (Table 4). E2 is known to influence the ECM in the uterus and vaginal tissues [88], in osteoblasts [89], and in the skin [90]. Interestingly, the overall directionality of the pathway as indicated by the median change, was opposite (negative) compared to the directionality of the pathway in the TGF-β1 and TGF-β1 + E2 groups (positive, Table 4). This is consistent with a study that found that E2 inhibited TGF-β1-induced ECM production in human and rat mesangial cells through GPER1 activation [22]. Of note is the repression of MMP14 and MMP2 as another study showed that E2 decreased MMP2-, MMP13-, and MMP14-mediated tissue matrix destruction [91]. These results are consistent with the significant reduction of *CLIC3* mRNA expression by E2 (Table 3) which is known to regulate MMP14 [65, 66]. Future studies should delineate the precise role of each ESR in regulating genes involved in ECM turnover.

## Conclusions

In conclusion, we were not able to decipher an effect of E2 on TGF-β1-induced EMT, but we do report the novel observation that TGF-β1 inhibited *ESR1*, *ESR2*, and *GPER1* mRNA expression and ESR1 protein expression in BEAS-2Bs. We also report that E2 specifically down-regulated the expression of *CLIC3* and *RBP7* which have been associated with pathogenic mechanisms of pulmonary fibrosis. We further highlight cellular pathways involved in chromatin remodeling as novel and specific targets of E2 in bronchial epithelial cells and opposing actions of TGF-β1 and E2 signaling on genes involved in ECM turnover. Although these data do not explicitly indicate a protective role for E2 in pulmonary fibrosis, these results suggest that E2 influences pathways relevant to pulmonary fibrosis and highlights potential roles for E2 in the lung that may contribute to sex-specific differences.

## Additional file

Additional file 1: **Table S1**. Genes differentially expressed (FDR-corrected *p*-value < 0.05) in TGFB1 group compared to vehicle control group. **Table S2**. Genes differentially expressed (FDR-corrected *p*-value < 0.05) in TGFB1 + E2 group compared to vehicle control group. (XLS 479 kb)

## Abbreviations

ACTA2: Alpha smooth muscle actin
ACTB: Beta-actin
BEAS-2Bs: Bronchial epithelial cells
CDH1: E-cadherin
CDH2: N-cadherin
CLIC3: Chloride intracellular channel 3
CRABP4: Cellular retinoic acid-binding protein 4
CTGF: Connective tissue growth factor
DMSO: Dimethyl sulfoxide
DUSP6: Dual specificity phosphatase 6
E2: 17β-Estradiol
EMT: Epithelial to mesenchymal transition
ERCC: External RNA controls consortium
ESR: Estrogen receptor
ESR1: Estrogen receptor alpha
ESR2: Estrogen receptor beta
FN1: Fibronectin
FVC: Forced vital capacity
FVC%: Percent predicted forced vital capacity
GAPDH: Glyceraldehyde phosphate dehydrogenase
GPER1: G-protein coupled estrogen receptor
GSEA: Gene set enrichment analysis
IPF: Idiopathic pulmonary fibrosis
KCNQ1: Potassium voltage-gated channel subfamily Q member 1
MIQE: Minimum information for publication of qPCR experiments
MMP14: Matrix metalloproteinase 14
MMP2: Matrix metalloproteinase 2
MT1-MMP: Matrix metalloproteinase 14
MT-MMP: Membrane-type matrix metalloproteinase
NURF: Nucleosome remodeling factor
PDGF: Platelet-derived growth factor
RASAL1: RAS protein activator like 1
RBP7: Retinol binding protein 7
RPS13: Ribosomal protein S13
SMAD3: SMAD family member 3
SNAI1: Snail family transcriptional repressor 1
SPRY4: Sprouty RTK signaling antagonist 4
TGF-β1: Transforming growth factor beta1
UIP: Usual interstitial pneumonia
VIM: Vimentin
